# A long-term ketogenic diet in young and aged rats has dissociable effects on prelimbic cortex and CA3 ensemble activity

**DOI:** 10.3389/fnagi.2023.1274624

**Published:** 2023-12-14

**Authors:** Abbi R. Hernandez, Maya E. Barrett, Katelyn N. Lubke, Andrew P. Maurer, Sara N. Burke

**Affiliations:** ^1^Division of Gerontology, Geriatrics, and Palliative Care, Department of Medicine, The University of Alabama at Birmingham, Birmingham, AL, United States; ^2^Department of Psychology, The University of Tennessee, Knoxville, Knoxville, TN, United States; ^3^Department of Neuroscience, McKnight Brain Institute, and Center for Cognitive Aging and Memory, University of Florida, Gainesville, FL, United States

**Keywords:** aging, ketosis, *Arc*, *Homer*, behavior

## Abstract

**Introduction:**

Age-related cognitive decline has been linked to distinct patterns of cellular dysfunction in the prelimbic cortex (PL) and the CA3 subregion of the hippocampus. Because higher cognitive functions require both structures, selectively targeting a neurobiological change in one region, at the expense of the other, is not likely to restore normal behavior in older animals. One change with age that both the PL and CA3 share, however, is a reduced ability to utilize glucose, which can produce aberrant neural activity patterns.

**Methods:**

The current study used a ketogenic diet (KD) intervention, which reduces the brain’s reliance on glucose, and has been shown to improve cognition, as a metabolic treatment for restoring neural ensemble dynamics in aged rats. Expression of the immediate-early genes *Arc* and *Homer*1a were used to quantify the neural ensembles that were active in the home cage prior to behavior, during a working memory/biconditional association task, and a continuous spatial alternation task.

**Results:**

Aged rats on the control diet had increased activity in CA3 and less ensemble overlap in PL between different task conditions than did the young animals. In the PL, the KD was associated with increased activation of neurons in the superficial cortical layers, establishing a clear link between dietary macronutrient content and frontal cortical activity. The KD did not lead to any significant changes in CA3 activity.

**Discussion:**

These observations suggest that the availability of ketone bodies may permit the engagement of compensatory mechanisms in the frontal cortices that produce better cognitive outcomes.

## 1 Introduction

In old age, increased activity in hippocampal CA3 is associated with worse cognitive outcomes (Yassa et al., 2011a; Maurer et al., 2017). Conversely, increased activation in the prelimbic cortex (PL) is correlated with better cognition (Hernandez et al., 2020b). While pharmacological agents have been shown to improve either PL (Banuelos et al., 2014) or CA3 function (Koh et al., 2010; Bakker et al., 2012, 2015; Robitsek et al., 2015), an effective therapeutic approach for optimizing neural activity patterns across these brain regions with divergent relationships between activation and cognitive performance has not been established.

Although the mechanisms of dysfunction are distinct between CA3 and PL, glucose utilization is disrupted in both of these structures with age (Gage et al., 1984; McNay and Gold, 2001; Gold, 2004; Pandya et al., 2016; Gardner et al., 2020). Moreover, blockade of glycolysis is sufficient to promote epileptogenesis (Samokhina et al., 2017), suggesting that impaired glucose metabolism can lead to aberrant activity patterns. While these data point to the potential of targeting energy metabolism to improve cognitive aging outcomes, enhancing insulin signaling has produced equivocal results regarding benefits to brain function (Moore et al., 2013; Barini et al., 2016; Anderson et al., 2017). In fact, age-related declines in glucose transporters within the brain (Ding et al., 2013; Hernandez et al., 2018b) suggest that targeting glycolysis will likely be insufficient to overcome neurometabolic deficiencies.

Rather than attempting to restore impaired glucose metabolism, an alternate mechanism for alleviating metabolic deficits in the brain is to shift energy metabolism from glucose to ketone body utilization. While consumption of a high fat diet that is also sufficiently high in carbohydrates, often referred to as a Western diet, is obesogenic and associated with poor health status (Rakhra et al., 2020), ketogenic diets restrict carbohydrate intake to levels low enough to induce nutritional ketosis. Ketosis is an alternate metabolic state that uses ketone bodies from fat to compensate when glycolysis is unavailable during fasting, intense exercise, or carbohydrate restriction. Remarkably, while glucose utilization is impaired in the brains of persons living with Alzheimer’s disease, ketone body utilization appears intact (Ogawa et al., 1996; Castellano et al., 2015). Moreover, a ketogenic diet in aged animals has been shown to reverse biochemical alterations within the PL and hippocampus that are specific to the unique disruptions of each region (Hernandez et al., 2018a,b). A ketogenic diet has also been shown to improve peripheral metabolism (Hernandez et al., 2020a), and enhance behavioral performance in both young and aged rats (Hernandez et al., 2018a) on a task that requires frontal-hippocampal interactions (Hernandez et al., 2017). Moreover, ketogenic diets alter the expression of signaling-related proteins within both the hippocampus and prefrontal cortex (Hernandez et al., 2018a) and gene expression within the hippocampus (Hernandez et al., 2019b). These data suggest that a ketogenic diet may normalize neural activity across CA3 and PL circuits that becomes disrupted in advanced age.

To test the hypothesis that cognitive enhancement in aged rats on a ketogenic diet is due to a normalization of neural activity across the frontal-medial temporal lobe circuit, the current study used single cell imaging of the mRNA for the immediate-early genes (IEGs) *Arc* and *Homer*1a (*H*1a) to measure ensemble dynamics in PL and CA3 in relation to behavior. *Arc and H*1a mRNA are transcribed immediately after neuronal activity related to attentive behavior, but intranuclear *Arc* appears within 5 min, while *H*1a is detected only after 30 min, due to large introns atypical of IEGs (Vazdarjanova et al., 2002). Cytoplasmic accumulation of these IEGs is similarly staggered, with cytoplasmic *Arc* appearing 30 min after activity (Guzowski et al., 1999) whereas cytoplasmic *H*1a is not detectable until after 60 min (Bottai et al., 2002; Marrone et al., 2008). Notably, the co-expression of *Arc* and *H*1a is high, with >95% of neurons positive for cytoplasmic *Arc* also showing nuclear *H*1a. Thus, monitoring *Arc* and *H*1a transcription permits the detection of activity during three distinct episodes at approximately 60, 30 min and immediately prior to sacrifice (Marrone et al., 2008). In the current experiment, young and aged rats performed two different behavioral tasks for 5 min each separated by 20 min and cytoplasmic *H*1a was used to infer activity levels while the rats were resting in the home cage prior to behavior (baseline). This enabled the evaluation of ensemble overlap across behavioral states (active vs. rest) and tasks.

## 2 Materials and methods

### 2.1 Subjects and handling

Fischer 344 × Brown Norway F1 (FBN) Hybrid male and female rats from the National Institute on Aging colony at Charles River were used in this study, as these rats are a robust model of non-pathological aging (McQuail and Nicolle, 2015; Hernandez et al., 2020c). A total of 36 total young (4–7 months) and aged (20–23 months) rats were split across control (*n* = 6 young male, *n* = 9 aged male, *n* = 1 young female and *n* = 2 aged female) and ketogenic (*n* = 8 young male, *n* = 8 aged male, *n* = 1 young female, and *n* = 1 aged female) diet groups. Additional females were unavailable for inclusion in this study, which precluded the analysis of any potential sex differences. The data from the female rats, however, did not vary from the means of the male rats and were thus included in the analysis. Moreover, similar results were obtained if the female rats were excluded. Because sex alone is not an adequate reason to exclude animals from a sample, the female rats were included. All rats were housed individually and maintained on a 12-h reversed light/dark cycle with all behavioral testing occurring in the dark phase. Note that reported ages correspond to the age of the rats upon arrival at the University of Florida vivarium. All rats were on the diet for 3 months prior to starting shaping on behavioral tasks, which took approximately 3 months. Thus, at the time of tissue collection young rats were 10–13 months and aged rats were 26–29 months. Mortality rates for this strain of rat begin to increase at 30 months (Fernandes et al., 1997). Therefore, to minimize age-related attrition we wanted to complete 3 months of the diet, plus the several months of training prior to 30 months of age. Additionally, it has been reported that Fischer 344 × Brown Norway hybrid rats start to show physical impairments at 28 months of age (McQuail and Nicolle, 2015). Because the WM/BAT task required rats to ambulate around a track, it was critical to begin cognitive testing prior to the emergence of sensorimotor impairments.

### 2.2 Diet

All rats were fed either a high-fat, low-carbohydrate KD (75.85% fat, 20.12% protein, 3.85% carbohydrate; Lab Supply; 5722, Fort Worth, TX, USA) or a calorically and micronutrient comparable control diet (CD; 16.35% fat, 18.76% protein, 64.89% carbohydrate; Lab Supply; 1810727, Fort Worth, TX, USA). The specific details on composition of the diets have been reported previously (Hernandez et al., 2018a,b). The primary fat source in the KD was a >95% C8 medium chain triglyceride (MCT; Neobee 895, Stephan, Northfield, IL, USA). Rats were weighed daily and given ∼51 kCal for males and ∼43 kCal for females at the same time each day for the first 12 weeks of the diet. Following a 12-week adjustment period to the diet and confirmation of sustained nutritional ketosis, the amount of food was restricted to reach an ∼15% reduction in body weight to motivate participation during behavioral testing. Access to water was available *ad libitum* for all animals.

### 2.3 Behavioral training and testing

**FIGURE 1:**
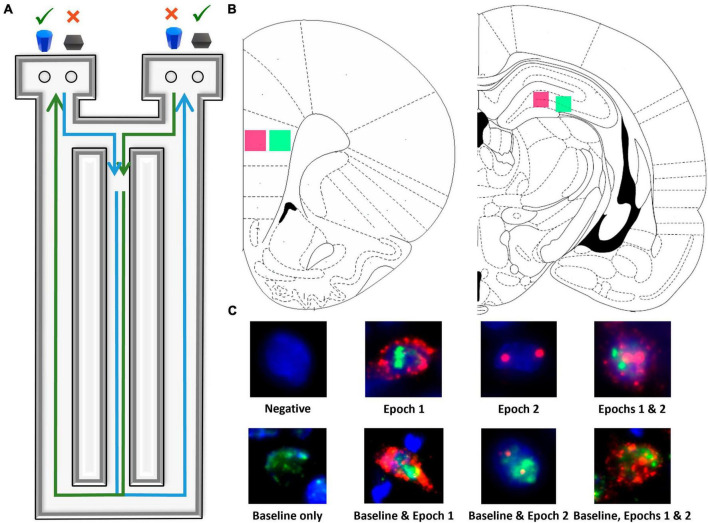
Image acquisition and analysis methodology. **(A)** Birds eye view of the figure-8 shaped maze used for all behavioral training and testing. **(B)** Regions imaged within superficial (pink) and deep (green) prelimbic cortex as well as proximal (pink) and distal (green) CA3. **(C)** Example cells of each level of activity (see section “2 Materials and methods” for further details).

All behavioral data for shaping, continuous alternations, object discrimination testing, and the working memory/bi-conditional association task for the animals in which neurobiology data are reported in the current study were documented in a previous publication (Hernandez et al., 2018a). Briefly, all behavioral testing occurred on a Figure 8-shaped maze (see Figure 1A) that was 67.5 inches long and 25 inches wide. The maze was constructed from wood and sealed with waterproof white paint. The center arm was made of clear acrylic to monitor gait (data not reported). The choice platforms each contained two food wells (2.5 cm in diameter) that were recessed into the maze floor by 1 cm. All arms were 4 inches wide. The choice platforms were contained within 7.5 cm raised walls and the right arm was contained within 20 cm high raised halls, but the center and left arms did not have walls.

During shaping, rats were placed on the maze for 10 min per day for two consecutive days to habituate to the maze. Food rewards (macadamia nuts, Mauna Loa, Keaau, Hawaii) were scattered throughout the maze to encourage exploration. Macadamia nuts, used for rats on both diets, were chosen based on their palatability and macronutrient composition (80% fat, 13.33% carbohydrates, and 6.67% protein), which would not inhibit ketosis. Importantly, the macadamia nuts did not interfere with the consumption of the control diet nor lead to weight gain, nor did they induce ketosis in the CD group. Rats were then trained to continuously alternate between the two outside arms of the maze in a Figure 8 motion, returning through the center between trials until they correctly alternated on at least 16 of 20 trials on two consecutive days.

Following alternation training, rats were trained to perform simple object discriminations both with and without alternations. These tasks did not have the bi-conditional object-in-place rule and were used to train the animals in the procedural aspects of moving an object to obtain a reward. Rats performed 20 trials/day of this task until a criterion of at least 80% correct was reached on two consecutive days. Rats were then tested on the cognitive dual task which combined spatial alternations with a biconditional association task (WM/BAT) that required the animal to continue to alternate, while also associating a particular object of a pair with the different testing platforms (see Figure 1A). Specifically, rats were presented with the same two objects in both arms with each object being the correct choice in opposing arms. This task requires working memory to complete the continuous alternation while also imposing an interrupting bi-conditional association task between alternations to increase the cognitive load. Should a rat mistakenly enter the wrong arm (for example, entering into the right arm following a right arm trial), the trial was recorded as a working memory error and rats were not presented with the object discrimination problem. All rats completed 20 trials/day until they reached a criterion performance of better than 80% correct on two consecutive days and then a final shorter session was run, and tissue was collected. Rats were trained to a common criterion to ensure that differences in the expression of *Arc* and/or *Homer*1a were due to age and/or diet rather than differences in performance or the number of rewards obtained.

### 2.4 Tissue collection and *Arc*/*Homer*1a catFISH labeling

On the final day of behavior, the testing session was comprized of two behavioral epochs (approximately 5 min each in duration), counterbalanced across rats. Rats completed yoked trials so that young and aged rats performed a comparable number of trials. To do so, an aged rat performed as many trials as possible within 5 min. The next young rat completed the same number of trials, or 5 min total, whichever came first. Following a 20-min rest in their home cage, rats performed the second epoch of behavior. Rats were then immediately placed into a bell jar containing isoflurane-saturated cotton (Abbott Laboratories, Chicago, IL, USA) separated from the animal by a wire mesh shield. Animals rapidly lost the righting reflex (<30 s), after which they were immediately euthanized via rapid decapitation. Tissue was extracted and flash frozen in 2-methylbutane (Acros Organics, NJ, USA) chilled in a bath of dry ice with 100% ethanol (∼−70°C). Tissue was stored at −80°C until cryosectioning. Prior to cryosectioning, one hemisphere from each experimental group was blocked so that tissue from different groups would be sliced together and so each slide would contain all experimental groups to control for potential variability is staining across slides. Sectioning was performed at 20 μm on a cryostat (Microm HM550) and thaw mounted on Superfrost Plus slides (Fisher Scientific). Sliced tissue was stored at −80°C until *in situ* hybridization.

*In situ* hybridization was performed to label *Arc* and *Homer*1a mRNA for catFISH (Vazdarjanova et al., 2002; Marrone et al., 2008) using commercial transcription kits and RNA labeling mix (REF #: 11277073910, Lot #: 26591021 and REF #11426346910:, Lot # 24158720; Roche Applied Science) to generate digoxigenin-labeled and fluorescein-labeled riboprobes using a plasmid template containing cDNA. Tissue was incubated in the generated probes overnight and labeled with an anti–digoxigenin-HRP conjugate or anti-fluorescein- (Ref#: 11207733910, Lot # 43500600 and REF#11426346910; LOT#45220020; Roche Applied Science). Fluorescein (Fluorescein Direct FISH; PerkinElmer, REF# 14921915, Lot #: 2587974) and Cy3 (TSA Cyanine 3 Tyramide; PerkinElmer, REF#: 10197077, Lot #: 2629833) were used to visualize labeled cells, and nuclei were counterstained with DAPI (Thermo Scientific).

### 2.5 *Arc* and *Homer*1a imaging and quantification

Fluorescence microscopy on a Keyence BZX-810 digital microscope (Keyence Corporation of America, Itasca, IL) was utilized to obtain z-stacks at 1 μm increments from 3 sections per region of interest for each rat. A total of 4 regions were imaged: deep and superficial prelimbic cortex (PL) and distal (CA3b) and proximal CA3 (CA3c), for a total of 12 images per rat (see Figure 1B). Because of the potential for conflating CA3a for CA2, this subregion of CA3 was not analyzed. Deep and superficial layers of PL were analyzed separately due to their distinct patterns of connectivity. Specifically, superficial PL layers are more reciprocally connected to other cortical areas, such as the perirhinal cortex (Burwell, 2000; Furtak et al., 2007; Agster and Burwell, 2009), while deep layers of PL are more connected to subcortical structures such as the nucleus reuniens (Vertes, 2002; Vertes et al., 2006; Jayachandran et al., 2019). A custom ImageJ plugin (available upon request) was utilized to segment nuclei and to classify cells as having no mRNA present, being positive for *Arc*, *Homer1*, or both, as well as the subcellular location of the mRNA. All experimenters analyzing cellular imaging data were blind to age, diet, and behavioral task order (WM/BAT vs. alternation first). As in previous studies (e.g., Maurer et al., 2017; Hernandez et al., 2018c), only fully visible cells within the median 20% of the optical planes were considered.

Figure 1C shows examples of all possible cell classifications and the temporal activity profile inferred from the cellular location of *Arc* and *Homer*1a mRNA. To summarize, cells were classified as one of the following: (1) negative for any staining, indicating no activity 60 min prior to sacrifice, (2) *Arc* cytoplasm + *Homer*1a foci, indicating activity during epoch 1 only, (3) *Arc* foci, indicating activity during epoch 2 only, (4) *Arc* cytoplasm + *Homer*1a foci + *Arc* foci, indicating activity during both epochs, (5) *Homer*1a cytoplasm, indicating home cage activity only, (6) *Homer*1a cytoplasm + *Arc* cytoplasm + *Homer*1a foci, indicating home cage and epoch 1 activity, (7) *Homer*1a cytoplasm + *Arc* foci, indicating home cage and epoch 2 activity, and (8) *Homer*1a cytoplasm + *Arc* cytoplasm + *Homer1* foci + *Arc* foci, indicating activity in the home cage and during both epochs.

To control for differences in activity between regions similarity scores (Vazdarjanova and Guzowski, 2004) were calculated across the two behavioral epochs as follows: $\frac{B-\left(E\,1\times E\,2\right)}{\frac{\left(E\,1+E\,2\right)}{2}-\left(E\,1\times E\,2\right)}$ where B is the proportion of cells active during both epochs of behavior and E1 and E2 are the proportion of cells active during the first or second behavioral epoch, respectively. If identical populations of cells were active during the two behavioral epochs, the subject would receive a value of 1. Conversely, a value of 0 indicates two entirely statistically independent populations of cells were activated during the two behavioral epochs. These values allow for the comparison across brain regions with different neural activity patterns by reducing each region to a single value, rather than information from multiple epochs of behavior.

### 2.6 Statistical analysis

Differences in neuronal activation during the behavioral epochs in relation to age, cortical layers and diet group were statistically evaluated by calculating the mean percentage of *Arc* positive cells per rat as done previously (Hartzell et al., 2013; Hernandez et al., 2018c,2020b) to avoid inflating statistical power by including multiple measures from one animal and ensure data do not violate the assumption of independent observations (Aarts et al., 2014).

Potential effects of age, layers/subregion and task were examined using repeated measures ANOVAs (ANOVA-RM) with the within-subject factors of behavioral task, cortical layers, pre-active status, and the between-subjects factors of age and diet. All analyses were performed with the Statistical Package for the Social Sciences v25 (IBM, Armonk, NY, USA) or GraphPad Prism version 9.1.0 for Windows (GraphPad Software, San Diego, CA, USA).^1^ Statistical significance was considered at p-values less than 0.05.

## 3 Results

### 3.1 Home cage associated *Homer*1a expression in prelimbic cortex and CA3 prior to behavior

**FIGURE 2:**
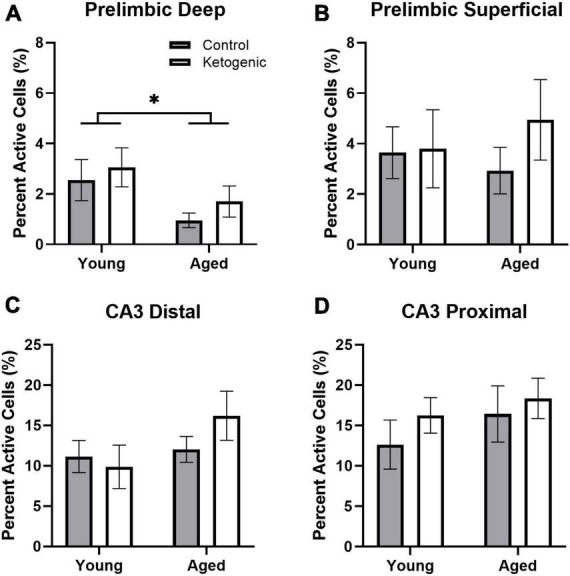
Baseline activity during home cage rest prior to behavior. **(A)** Aged rats had significantly decreased baseline activity within the deep layers of the prelimbic cortex. **(B)** There were no age or diet effects within the superficial layers of PL. **(C,D)** Age did not significantly influence baseline activity within distal or proximal CA3. There was no significant effect of diet on baseline *Homer*1a expression within any region or layer. Data represent group means ± 1 SEM. * indicates *p* < 0.05.

Figure 2 shows the percent of neurons that were positive for *Homer*1a in the cytoplasm, indicating activation prior to behavior during baseline for PL (Figures 2A, B) and CA3 (Figures 2C, D). The proportion of cells active at baseline was significantly different between the PL cortex and CA3 subregions [*F*_(3,96)_ = 44.15; *p* < 0.001], with more baseline activity within both subregions of CA3 compared to PL. This was not significantly affected by age [*F*_(1,32)_ = 1.43; *p* = 0.24] nor diet [*F*_(1,32)_ = 1.79; *p* = 0.19], and none of these factors significantly interacted (*p* > 0.24 for all comparisons).

Within the PL, there was significantly more baseline activity within the superficial layers than there was in the deep layers [*F*_(1,32)_ = 7.87; *p* = 0.008]. This did not significantly vary by age [*F*_(1,32)_ = 0.59; *p* = 0.45] or diet group [*F*_(1,32)_ = 1.09; *p* = 0.31] (Figures 2A, B). Furthermore, there were no significant interaction effects between age, diet, and cortical layers (*p* > 0.18 for all comparisons). Within the deep layers, however, there were significantly fewer neurons that had *Homer*1a in the cytoplasm in aged compared to young rats [*F*_(1,32)_ = 5.72; *p* = 0.02]. There was not a significant effect of age on baseline activity within the superficial layers [*F*_(1,32)_ = 0.03; *p* = 0.87].

The effect of subregion within CA3 on baseline activity did not reach statistical significance, though there was a trend toward greater proportion of active cells within proximal CA3 relative to distal CA3 [*F*_(1,32)_ = 3.68; *p* = 0.06; Figures 2B–D]. There were no differences across age [*F*_(1,32)_ = 2.79; *p* = 0.10] or diet [F_(1,32)_ = 1.15; *p* = 0.29] groups. Furthermore, age and diet did not significantly interact with each other [*F*_(1,32)_ = 0.23; *p* = 0.63], nor with cortical layer (*p* > 0.72 for both comparisons).

### 3.2 Behavioral performance and nutritional ketosis

**FIGURE 3:**
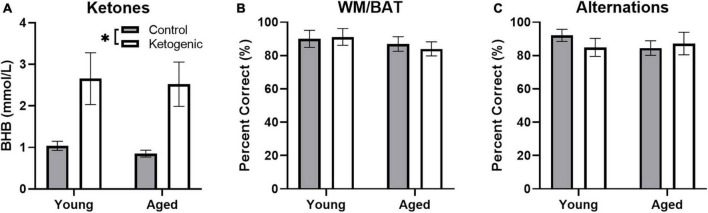
Behavioral performance and β-hydroxybutyrate (BHB) levels across age. **(A)** Production of β-hydroxybutyrate (BHB), a major circulating ketone body, did not differ across age groups, but was significantly elevated in rats fed a ketogenic diet relative to a control diet. On both the **(B)** WMBAT and **(C)** alternation task young and aged rats reached criterion performance levels and did not show a significantly different percent of correct trials in either the control or ketogenic diet conditions on the final day of testing. Data represent group means ± 1 SEM. * indicates *p* < 0.05.

Blood was collected during sacrifice to measure peripheral β-hydroxybutyrate (BHB; a major circulating ketone body) immediately following behavioral testing. Rats fed a ketogenic diet had significantly more BHB than control-fed rats [*F*_(1,34)_ = 17.08; *p* = 0.0002; Figure 3A]. There was no significant effect of age [*F*_(1,34)_ = 0.16; *p* = 0.69] nor did age significantly interact with diet [*F*_(1,34)_ = 005; *p* = 0.95], suggesting that young and aged rats had reached similar levels of ketosis.

As reported in a previous study (Hernandez et al., 2018a), both young and aged rats show improved performance on a spatial alternation and the working memory/bi-conditional association task (WM/BAT) when on a long-term ketogenic diet compared to animals on a calorie-matched control diet. On the final day of behavioral testing day, in which IEG expression was quantified, all rats performed two 5 min epochs of behavior separated by 20 min. In counterbalanced order, rats performed the WM/BAT task and continuous spatial alternations on the Figure 8 maze. While there were initial age-related differences and performance improvements related to the ketogenic diet (Hernandez et al., 2018a), all rats were previously trained to a criterion performance. Thus, on the final day of testing, there were no significant differences across age group [*F*_(1, 32)_ = 1.17; *p* = 0.29] or diet condition [*F*_(1, 32)_ = 0.04; *p* = 0.85] on WM/BAT performance accuracy, nor did these factors significantly interact [*F*_(1, 32)_ = 0.19; *p* = 0.67; Figure 3B]. Similarly, performance accuracy for continuously alternating on the maze did not significantly differ between age [*F*_(1, 32)_ = 0.24; *p* = 0.63] or diet [*F*_(1, 32)_ = 0.18; *p* = 0.68] groups. Moreover, there was not a significant interaction between age and diet on alternation performance [*F*_(1, 32)_ = 0.86; *p* = 0.36; Figure 3C].

### 3.3 Age and diet influenced behaviorally induced activity within superficial, but not deep, prelimbic cortex

**FIGURE 4:**
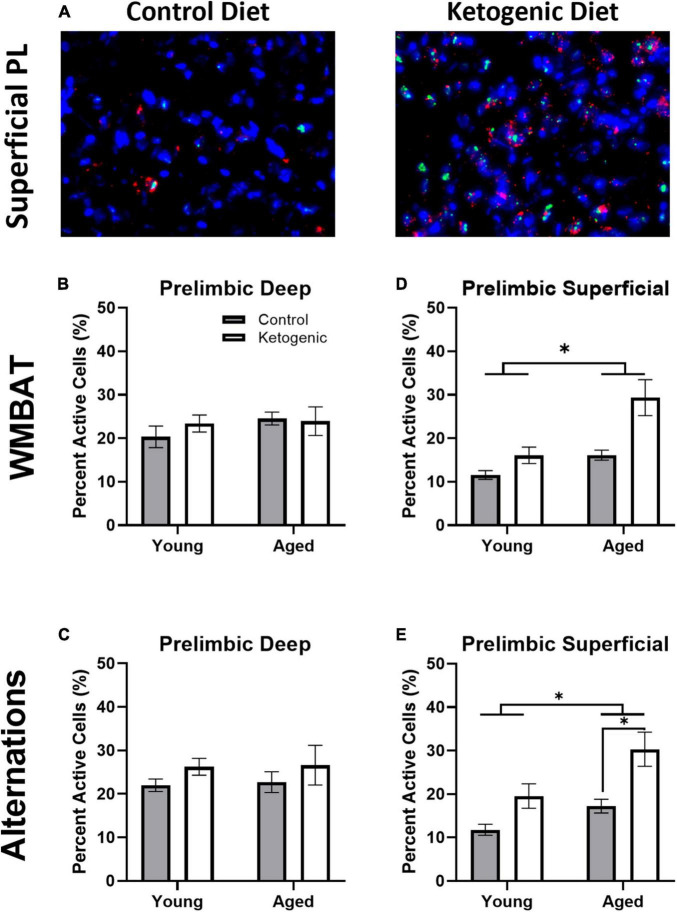
The ketogenic diet increased cellular activity in the superficial, but not deep, prelimbic cortex. **(A)** Representative images from the superficial layer of the prelimbic cortex in aged rats on the control (left) and ketogenic (right) diets. **(B,C)** While there was no significant influence of age, diet nor task within the deep layers of the PL, **(D,E)** both age and diet significantly affected activity within the superficial layers. Data represent group means ± 1 SEM. * indicates *p* < 0.05.

Figure 4A shows representative images of *Arc* and *Homer*1a expression in superficial layers of PL for aged rats on a control (left panel) or ketogenic diet (right panel). The percent of cells active during the two behavioral epochs did not significantly differ between WM/BAT and the continuous alternation tasks within either the deep [*F*_(1,32)_ = 0.59; *p* = 0.45; Figures 4B, C] or superficial [*F*_(1,32)_ = 1.81; *p* = 0.19; Figures 4D, E] layers of PL. Both age [*F*_(1,32)_ = 13.39; *p* = 0.001] and the ketogenic diet [*F*_(1,32)_ = 17.20; *p* < 0.001] significantly increased activity within the superficial layers, but neither age [*F*_(1,32)_ = 0.52; *p* = 0.48] nor diet [*F*_(1,32)_ = 1.70; *p* = 0.20] significantly altered activity within the deep layers. However, *post-hoc* analyses indicated that there was a significantly greater degree of activation in aged KD-fed rats than young KD-fed rats [*t*_(32)_ = 3.91; *p* = 0.003], but this was not true for young vs. aged control-fed rats [*t*_(32)_ = 1.31; *p* = 0.73]. There were no significant interactions between task, age, or diet for the deep or superficial layers of PL (*p* > 0.14 for all comparisons). The observation that a KD affected activation patterns in superficial but not deep cortical layers of PL, suggests that neurons that project to other cortical structures are more likely to be affected by interventions that elevate ketone bodies in the brain than are neurons that project to subcortical areas.

### 3.4 Task, but not diet, influenced behaviorally induced activity within CA3

**FIGURE 5:**
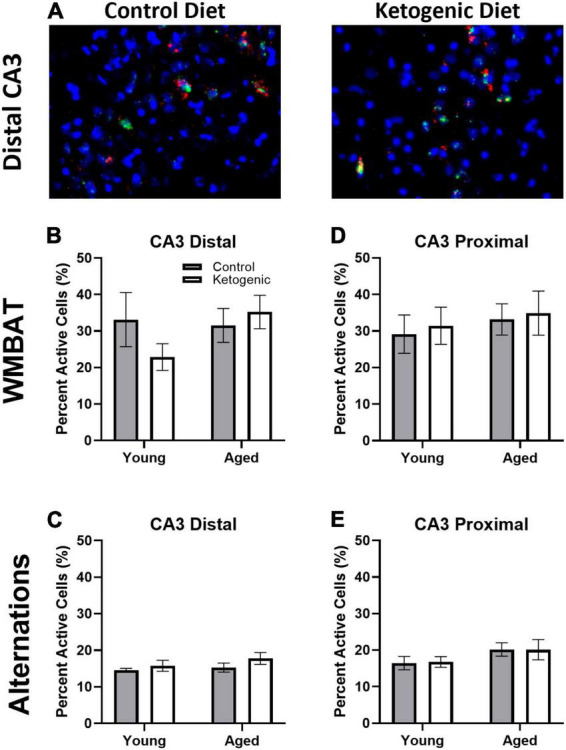
Task type influenced cellular activity within CA3. **(A)** Representative images from the distal portion of CA3 in aged rats on the control (left) or ketogenic (right) diets. **(B–E)** Activity was significantly greater during the WMBAT task relative to the alternation task within both regions of CA3 examined. However, there was no significant effect of age or diet on activity within this region. Data represent group means ± 1 SEM.

Figure 5A shows representative images of *Arc* and *Homer*1a expression in distal CA3 for aged rats on a control (left panel) or ketogenic diet (right panel). In contrast to the PL, the percent of cells active during the two behavioral epochs was significantly higher during WM/BAT compared to continuous alternations within both distal [*F*_(1,32)_ = 39.28; *p* < 0.001; Figures 5B, C] and proximal [*F*_(1,32)_ = 28.72; *p* < 0.001; Figures 5D, E] subregions of CA3. There was a trend for aged rats to have a higher proportion of active neurons during behavior compared to the young rats [*F*_(1,64)_ = 2.71, *p* = 0.1]. However, there was no significant effect of diet within distal or proximal CA3 during either task (*p* ≥ 0.18 for all comparisons). These observations suggest that more CA3 neurons are active when the cognitive load of the task increases and that aged rats tended to have higher levels of CA3 activity regardless of diet condition.

### 3.5 Population overlap across behavioral tasks is changed with age in prelimbic cortex but not CA3

**FIGURE 6:**
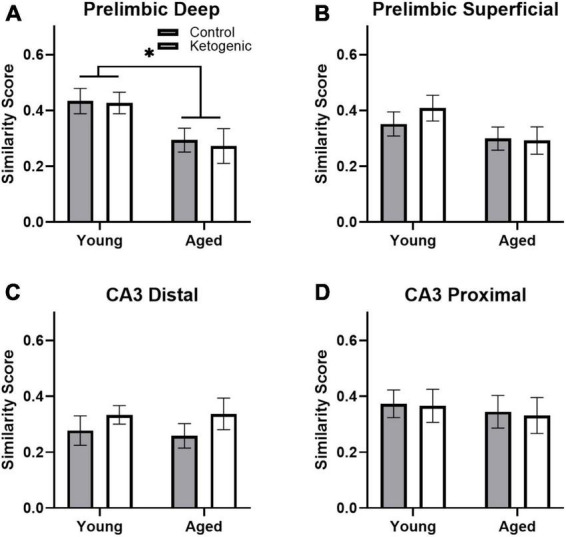
Similarity scores, representing ensemble activity overlap, were only affected by age within PL. **(A)** Aged rats had significantly lower ensemble activity overlap within the deep, **(B)** but not superficial layers of the PL. There were no differences in similarity score across diet groups in any region, nor did age significantly affect similarity in ensemble activity within **(C)** distal or **(D)** proximal CA3. Data represent group means ± 1 SEM. * indicates *p* < 0.05.

A similarity score was calculated for each layer of the prelimbic cortex separately for each rat as done previously (e.g., Vazdarjanova and Guzowski, 2004; Burke et al., 2005; Hernandez et al., 2018c). There was no difference in similarity score across the superficial and deep layers of PL [*F*_(1,32)_ = 0.93; *p* = 0.34; Figures 6A, B]. There was, however, a significant decrease in similarity scores in aged rats relative to the young group [*F*_(1,32)_ = 7.19; *p* = 0.01]. Diet did not significantly impact similarity score [*F*_(1,32)_ = 0.02; *p* = 0.90], and there were no significant interactions effects between diet, age, and PL layers (*p* > 0.11 for all comparisons). These data replicate a previous observation that aged rats have reduced population overlap across two different tasks in the same spatial context compared to young animals (Hernandez et al., 2018c). Because PL neurons has been observed to fire in association with common features across different episodes (Morrissey et al., 2017), these data suggest that PL neural ensembles in older animals are less able to bridge common elements across distinct but overlapping episodes and this is not reversed by diet. In CA3, there was no difference in similarity score between distal and proximal subregions [*F*_(1,32)_ = 2.30; *p* = 0.14; Figures 6C, D]. Furthermore, the similarity score for CA3 was not significantly affected by age [*F*_(1,32)_ = 0.22; *p* = 0.65] or diet [*F*_(1,32)_ = 0.46; *p* = 0.50]. Finally, there were no significant interactions between subregion, age, or diet on similarity score (*p* > 0.26 for all comparisons).

### 3.6 Cells with baseline activity are more likely to show activity during behavior

**FIGURE 7:**
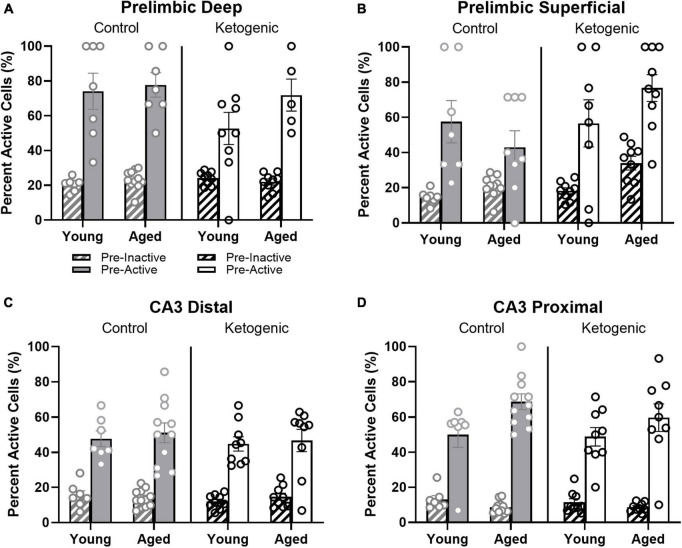
Pre-active cells were more likely to be active during behavior than pre-inactive cells. **(A,B)** While neurons that were active in the home cage prior to behavior (pre-active cells) were more likely to be active within both deep and superficial layers of the PL than cells that were quiescent prior to behavior (pre-inactive), diet significantly influenced the likelihood that pre-active cells would again be active during behavior within the superficial, but not deep layers of PL. In **(C,D)** CA3, there was also a significant bias for pre-active to have an increased probably of also having activity during behavior compared to the pre-inactive cells. This did not vary as a function of diet condition. The pre-active bias, however, was significantly greater in proximal compared to distal CA3. Within proximal CA3, this bias was also significantly greater for young compared to aged rats.

Previous studies have reported that neuronal populations with higher firing rates are more likely to be active across different behavioral states and contexts than cells with lower firing rates (Mizuseki and Buzsáki, 2013; Buzsáki and Mizuseki, 2014; Grosmark and Buzsaki, 2016; Witharana et al., 2016). This is hypothesized to reflect a skewed or lognormal excitability distribution of neural activity (Buzsáki and Mizuseki, 2014; Grosmark and Buzsaki, 2016). To examine the extent that age and diet may alter the skewed excitability distribution of brain organization, the behavior-related activity profiles of neurons that were active or inactive during baseline in the home cage were compared. In other words, the proportion of cells active at baseline (that is, “pre-active” cells) that were also active during one or more behavioral epochs were compared to the proportion of cells inactive at baseline (that is, “pre-inactive” cells) that were active during one or more behavioral epochs. Within the PL, pre-active cells were significantly more likely to have activity during behavior than pre-inactive cells [*F*_(1,52)_ = 123.72; *p* < 0.001; Figures 7A, B] and this behavioral-related activity bias of pre-active cells did not significantly interact with the cortical layers [*F*_(1,52)_ = 1.62; *p* = 0.21], age groups [*F*_(1,52)_ = 0.004; *p* = 0.95], or diet conditions [*F*_(1,52)_ = 0.20; *p* = 0.66]. This observation suggests that both the deep and superficial layers of the PL follow a skewed excitability distribution that is not altered by age or diet. Finally, there was a significant interaction between diet and PL layer [*F*_(1,52)_ = 5.82; *p* < 0.02], such that the ketogenic diet was associated with greater activation in the superficial [*F*_(1,28)_ = 4.63; *p* < 0.05], but not deep cortical [*F*_(1,24)_ = 1.60; *p* = 0.22] layers of PL. The observation that the ketogenic diet increases activity in superficial but not deep layers of the PL is consistent with what was observed when baseline activity was not accounted for (Figure 4).

Within distal and proximal CA3, pre-active cells were significantly more likely to be active during behavior than pre-inactive cells [*F*_(1,64)_ = 360.59; *p* < 0.001; Figures 7C, D]. The increased probability for pre-active cells to also show activity during behavior compared to neurons without baseline activity significantly differed between distal and proximal CA3 [*F*_(1,64)_ = 9.27; *p* < 0.003], with proximal CA3 having a larger difference in behavioral-related activity between baseline active and inactive cells than distal CA3. Different patterns of anatomical input to distal vs. proximal CA3 could contribute to this observed difference in activity patterns (Witter, 2007; Lee et al., 2015, 2021, 2022). For example, distal CA3 receives more direct input from the entorhinal cortex while proximal CA3 receives mossy fiber input from both the supra- and infrapyramidal blades of the dentate gyrus (Witter, 2007). The increased probability for pre-active cells to also show activity during behavior compared to neurons without baseline activity also significantly differed between age groups [*F*_(1,64)_ = 5.75; *p* < 0.02], with older rats having a greater difference between cells that showed activity prior to behavior vs. those cells that were quiescent in the home cage. In other words, aged CA3 pyramidal cells that had activity in the home cage before behavior (i.e., pre-active) were more likely to fire during behavior than were young pre-active CA3 cells. This observation is consistent with previous reports of elevated activity within aged CA3 neurons relative to young animals (Wilson et al., 2005; Thomé et al., 2016; Maurer et al., 2017; Lee et al., 2021). The behavioral-related activity bias of pre-active neurons did not significantly interact with diet [*F*_(1,64)_ = 0.60; *p* = 0.42]. This suggests that aged CA3 neurons have activity dynamics that are more likely to not change across different behavioral states compared to young CA3 neurons, which is consistent with what has been observed from electrophysiological recordings (Wilson et al., 2005, 2006; Lee et al., 2022). Importantly, diet condition did not have any significant effects on the pre-active vs. pre-inactive bias, nor did it interact with age or CA3 subregion (*p* > 0.22 for all comparisons). This observation is consistent with a previous finding that the ketogenic diet did not alter the expression of genes that were related to synaptic transmission within CA3 (Hernandez et al., 2019b).

### 3.7 Pre-active, but not behaviorally induced, cellular activity correlates with behavioral performance

Simple linear regression was used to test whether cellular activity before or during behavioral performance correlates with task acquisition across diet groups. Activity within the superficial prelimbic cortex was investigated, as this is the region in which differences were observed across diet groups. Behavioral performance on days 12–15 of training was used to investigate this potential relationship, as this was the time frame in which rats on a ketogenic diet consistently outperformed rats on the control diet (Hernandez et al., 2018a).

**FIGURE 8:**
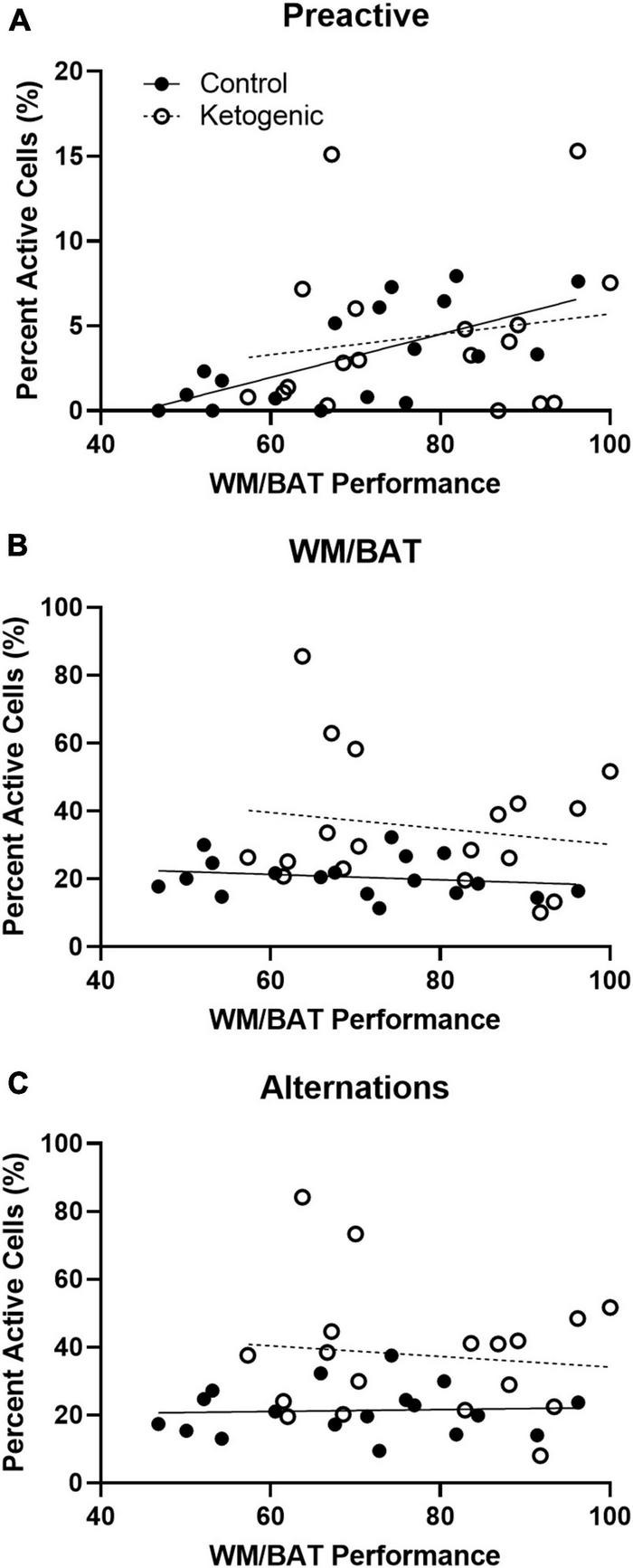
Pre-active cellular activity correlated with behavioral performance in control-fed, but not ketogenic-fed, rats. **(A)** Pre-activity within the superficial layers of the PL correlate with WM/BAT task performance for control-fed but not ketogenic-fed rats. Behaviorally induced cellular activity did not correlate with task performance during **(B)** WM/BAT nor during **(C)** alternations for either diet group.

For control-fed rats, task performance significantly correlated with the percent of pre-active cells within the superficial PL [*R*^2^ = 0.42; *F*_(1,16)_ = 11.64; *p* < 0.01 when adjusted for multiple comparisons] (Figure 8A). However, task performance did not significantly correlate with the percent of pre-active cells within the superficial [*R*^2^ = 0.03; *F*_(1,16)_ = 0.53; *p* = 0.48] layers of the PL in the ketogenic diet-fed group. Together these data indicate that higher levels of baseline activity in the superficial PL are associated with better cognitive performance under normal conditions. However, with elevated baseline PL activity following a ketogenic diet this relationship is no longer detectable, as all animals show higher activation and better cognitive performance. Cellular activity during task performance, for both WM/BAT and spatial alternations, did not significantly correlate with task performance during this time (*p* > 0.50 for both comparisons; Figures 8B, C).

## 4 Discussion

The present study utilized the cellular compartment analysis of temporal activity by fluorescence *in situ* hybridization (catFISH) for the immediate early genes (IEGs) *Arc* and *Homer*1a (Guzowski et al., 1999; Vazdarjanova et al., 2002) to quantify neuronal activity during three distinct epochs (Marrone et al., 2008): one prior to behavior, one while rats performed the working memory/biconditional association task (WM/BAT), and another while rats performed a continuous spatial alternation task. Activity was quantified in young and aged rats fed a ketogenic (KD) or calorically equivalent control diet (CD). The novel findings observed in the current study are that a long-term ketogenic diet increased the proportion of active neurons in the superficial layers of the PL in the aged rats. This indicates that elevating ketone bodies in old animals alters the ensemble dynamics of PL neurons that project to other cortical structures adding to an emerging understanding of how diet can directly affect brain function. Moreover, a significant correlation between pre-active cellular activity within the PL and WM/BAT task acquisition in control-fed rats was observed, with greater activity being associated with better performance. This correlation was not detected in the ketogenic diet-fed rats, as these rats showed greater baseline activation overall and better cognitive performance on this task.

Additionally, previous results were replicated regarding elevated IEG expression in aged CA3 neurons (Maurer et al., 2017), altered ensemble dynamics in PL (Hernandez et al., 2018c,2020b), and a bias for neurons that were active in the home cage prior to behavior to also be active during behavior compared to those cells that did not show activity in the home cage (Mizuseki and Buzsáki, 2013; Grosmark and Buzsaki, 2016). While it is possible that a ketogenic diet is capable of altering some aspect of IEG transcription kinetics, the replication of previous results along with the absence of brain-wide alterations in *Arc* of *Homer*1a expression indicates IEG responsiveness to typical activation remains intact. However, it is possible that there are specific diet-related alterations in neuronal activity in an uninvestigated brain region that is influencing the alterations in the PL presented here. Within the PL, there was also a reduction in the ensemble overlap for neurons that active during both WM/BAT and spatial alternations in aged compared to young rats, as previously observed (Hernandez et al., 2018c). This age difference was not affected by the KD, however. The KD also did not lead to any differences in activation within either distal or proximal CA3. Within CA3, the aged rats had an increase in the probability that a neuron that was active in the home cage prior to behavior would also be active during behavior compared to young rats. This was particularly evident in proximal CA3, but this age difference was not affected by the KD.

Previous studies have shown that a KD diet with medium chain triglyceride oil as the primary fat source is able to improve cognitive performance in both young and aged rats on both spatial alternations and WM/BAT (Hernandez et al., 2018a). Additionally, the KD leads to increased expression of vesicular GABA transporter in both the hippocampus and frontal cortex (Hernandez et al., 2018b), and alters the gut microbiome composition (Hernandez et al., 2022a,2018b). Importantly, biochemical effects of the KD have been reported to differ between the hippocampus and the frontal cortex. Specifically, in the hippocampus the age-related decrease in expression of the monocarboxylate transporter (MCT) 4, which is primarily on astrocytes, is restored to the levels of young animals by the KD (Hernandez et al., 2018b). In the frontal cortex, the age-related decrease in expression of MCT2, which is primarily on neurons, is restored to the levels of young animals by the KD (Hernandez et al., 2018a). The current findings add to this growing body of evidence that a systemic KD intervention has distinct effects on the frontal cortex and the hippocampus.

CA3 undergoes a number of alterations in advanced age that have been linked to impairments on hippocampus-dependent behaviors. These include changes in synapses (Villanueva-Castillo et al., 2017; Buss et al., 2021), gene expression (Haberman et al., 2011; Hernandez et al., 2019b), altered neuron firing patterns (Wilson et al., 2005; Robitsek et al., 2015; Thomé et al., 2016; Lee et al., 2021, 2022), and elevated activation (Yassa et al., 2011b; Maurer et al., 2017). One previous study that used Real-Time PCR to quantify the expression of genes related to synaptic transmission and plasticity reported that 3 months of a KD diet in young and aged rats did not lead to any significant changes in gene transcription within CA3. Thus, it is conceivable that age-related changes in CA3 synaptic transmission are not reversed by a KD (Hernandez et al., 2019b). The current data are consistent with this idea and extend it to suggest that age-related increases in CA3 excitability are also not affected by a long-term KD intervention. Notably, when whole hippocampus homogenates are analyzed, 3 months of a KD does lead to increased expression of the vesicular glutamate transporter (Hernandez et al., 2018b), and decreased expression of the protein rho-associated coiled-coil containing protein kinase 2 (ROCK2) (Hernandez et al., 2019b), which regulates cytoskeletal elements to modify spine stability and is associated with synaptic loss (Huentelman et al., 2009; Swanger et al., 2015). Thus, the effects observed from whole hippocampus homogenates must be due to changes in other hippocampus subregions. The dentate gyrus in particular appears to undergo significant changes in the transcription of synapse-related genes following 3 months of a KD (Hernandez et al., 2019b). Because advanced age is associated with reduced metabolic activity (Small et al., 2002, 2004) and lower levels of *Arc* expression in the dentate gyrus of aged compared to young animals (Small et al., 2004; Penner et al., 2011), future experiments should examine whether or not a KD can restore normal activity levels within the dentate gyrus of old animals. The sustained transcription of *Arc* in this region (Ramirez-Amaya et al., 2013), however, precludes the ability to determine the behavioral epoch that is associated immediate-early gene transcription. Thus, the *Arc*/*Homer*1a catFISH method is not possible in the dentate gyrus for the behavioral procedures used in the current experiments.

While activity within CA3 was not affected by the KD, the proportion of neurons that were active during behavior increased in the KD-fed rats in the superficial layers of the PL compared to animals on the control diet. While this occurred in both age groups, the increase in superficial layer neuron activation was particularly evident in the aged rats. In addition, cognitive outcomes within the control-fed group significantly correlated with the proportion of pre-active cells, but not behaviorally induced cells. This relationship was not observed in KD-fed rats, indicating that *long-term* alterations in cellular activity potentially drive behavioral differences across diet groups rather than short term differences in cell firing specifically during task performance. In other words, a chronic elevation of ketone bodies could alter the functional resting state network architecture of the superficial PL to bolster cognitive performance. These observations have implications for the potential mechanisms of how a KD intervention improves cognitive performance. A previous study reported that aged rats with greater activation the of PL neurons that projected to the perirhinal cortex had better performance on WM/BAT (Hernandez et al., 2020b). Relatedly, fMRI research with human study participants has shown that higher BOLD-signal values in the frontal cortices of older adults are associated with better cognitive performance (Cabeza et al., 2002, 2004; Lighthall et al., 2014). This suggests that in the face of age-related changes in medial temporal activity, compensatory activation of frontal cortices is engaged to maintain cognitive function. Because glucose utilization in the frontal cortices is disrupted in advanced age (Gage et al., 1984; Castellano et al., 2019), but ketone body metabolism remains intact (Croteau et al., 2018; Castellano et al., 2019), it is possible that a KD increases the availability of the ketone bodies β-hydroxybutyrate and acetoacetate for neuronal metabolism. Ketone body elevation may therefore support enhanced activation in the prelimbic cortices of aged animals. In other words, dietary ketosis may increase the energy supply to the frontal cortices enabling enhanced activation that can promote compensation, leading to better cognitive outcomes. Notably, it does not appear that the KD restores age-related changes in neural ensemble activity to the patterns that are observed in young animals. Alternatively, aged subjects may utilize different neural strategies for task completion than young subjects (Wiener et al., 2013; Lester et al., 2017; Newcombe et al., 2023).

Several limitations in the current experiments are worth mentioning. First, the experiments were not sufficiently powered to consider sex as a biological variable. While a small number of females were included, the lack of availability of female rats of the Fischer344 x Brown Norway hybrid strain during the time that these experiments were conducted precluded the ability to have matched sample sizes for both sexes. Other studies have reported sex differences in neuronal metabolism with age (Yao et al., 2011; Ding et al., 2013; Yoshizawa et al., 2014; Shang et al., 2020). It is therefore critical that future research evaluate the efficacy, metabolic, and other biochemical effects of a KD in males and females. Another caveat of the current data is that both diet groups were fed only once daily and underwent modest caloric restriction to encourage motivation on the appetitive-based behavioral testing. Due to this time-restricted feeding, both groups experienced intermittent fasting. Intermittent fasting, even with a normal control diet, can affect gene expression in the brain (Castrogiovanni et al., 2018; Spezani et al., 2020; Ng et al., 2022), confer neuroprotection (Halagappa et al., 2007; Mattson et al., 2017; Anton et al., 2018), and alter the gut microbiome (Hernandez et al., 2022a,b). Because all rats received intermittent fasting, it is possible that potential diet by age interactions were less than would have been observed with free feeding animals. In fact, rats the receive *ad libitum* feeding from middle- to old-age have worse cognitive performance compared to both ketogenic and control-fed rats that received intermittent fasting during the same time period (Hernandez et al., 2022b). Lastly, due to the extensive training rats received on the behavioral tasks, there may have been enrichment effects capable of enhancing cognitive function. In fact, training on this paradigm does result in an increase in rich-club organization of old rats on this task (Colon-Perez et al., 2019). However, despite differences in brain connectivity post-training in aged rats, these same rats did not demonstrate altered IEG expression in the frontal cortex.

In conclusion, a KD diet was associated with increased activation of neurons in the superficial layers of the PL. This may reflect the engagement of compensatory mechanisms for improving cognition in the presence of elevated ketone bodies, which can meet the energetic needs of the frontal cortices when glucose utilization is compromised. In contrast to PL, CA3 ensemble activation patterns were not affected by diet. Future work interrogating the effects of a ketogenic diet on neuronal activity within additional brain regions could further elucidate the role of diet and energy metabolism in age-related cognitive decline.

## Data availability statement

The raw data supporting the conclusions of this article will be made available by the authors, without undue reservation.

## Ethics statement

The animal study was approved by the University of Florida Institutional Animal Care and Use Committee (IACUC). The study was conducted in accordance with the local legislation and institutional requirements.

## Author contributions

AH: Conceptualization, Formal analysis, Funding acquisition, Investigation, Methodology, Supervision, Visualization, Writing – original draft, Writing – review and editing. MB: Data curation, Investigation, Writing – review and editing. KL: Data curation, Investigation, Project administration, Writing – review and editing. AM: Conceptualization, Writing – review and editing. SB: Conceptualization, Formal analysis, Funding acquisition, Methodology, Project administration, Resources, Supervision, Writing – review and editing, Writing – original draft.

## Footnotes

1
www.graphpad.com
